# The multi-step process of building TB/HIV collaboration in Cambodia

**DOI:** 10.1186/1478-4505-10-34

**Published:** 2012-10-18

**Authors:** Mao Tan Eang, Mean Chhi Vun, Khun Kim Eam, Samreth Sovannarith, Seng Sopheap, Ngauv Bora, Rajendra Yadav, Masami Fujita, Bernard Tomas, Massimo Ghidinelli, Pieter van Maaren, William A Wells

**Affiliations:** 1 National Centre for Tuberculosis and Leprosy Control, Phnom Penh, Cambodia; 2 National Center for HIV/AIDS, Dermatology and STDs (NCHADS), Phnom Penh, Cambodia; 3 World Health Organization, Phnom Penh, Cambodia; 4 World Health Organization, Western Pacific Regional Office, Manila, Philippines; 5 Pan American Health Organization, Washington DC, USA; 6 Independent Consultant,New York, USA

**Keywords:** Tuberculosis, HIV/AIDS, TB/HIV, Policy change

## Abstract

Tuberculosis and HIV/AIDS have synergistic health impacts in terms of disease development and progression. Therefore, collaborative TB and HIV/AIDS activities are a logical health systems response. However, the establishment of these activities presents a challenge for countries that have strong vertical disease programs that differ in their implementation philosophies. Here, we review the process by which TB/HIV collaboration was established in Cambodia. A cycle of overlapping and mutually reinforcing initiatives – local research; piloted implementation with multiple options; and several rounds of policy formulation guided by a cross-functional Technical Working Group – was used to drive nationwide introduction of a full set of TB/HIV collaborative activities. Senior Ministry of Health officials and partner organizations brought early attention to TB/HIV. Both national programs implemented initial screening and testing interventions, even in the absence of a detailed, overarching framework. The use of multiple options for HIV testing identified which programmatic options worked best, and early implementation and pilots determined what unanswered questions required further research. Local conduct of this research – on co-treatment timing and TB symptom screening – speeded adoption of the results into policy guidance, and clarified the relative roles of the two programs. Roll-out is continuing, and results for a variety of key indicators, including screening PLHIV for TB, and testing TB patients for HIV, are at 70-80% and climbing. This experience in Cambodia illustrates the influence of health research on policy, and demonstrates that clear policy guidance, the pursuit of incremental advances, and the use of different approaches to generate evidence can overcome structural barriers to change and bring direct benefits to patients.

## Background

Globally, tuberculosis (TB) and HIV/AIDS have proven to be a deadly and mutually reinforcing combination. Persons living with HIV (PLHIV) have 20–37 times greater risk of developing TB [1]; as a result, increasing HIV prevalence has led to a rapid rise in the number of TB notifications [2]. In turn, TB contributes to ~20% of the estimated deaths due to HIV infection [3].

Early efforts to address this threat were limited. Subsequently, however, the necessary TB/HIV activities were defined in the World Health Organization (WHO)’s 2004 interim policy [4] and accompanying guidelines [5]. Under this policy, both the TB and HIV programs are responsible for establishing the mechanisms for collaboration. The HIV program is more responsible for the 3Is: intensified case finding (ICF) of TB among PLHIV; isoniazid preventive therapy (IPT) for PLHIV with latent TB [6]; and infection control (IC) [7]. Finally, the TB program is more responsible for HIV testing and prevention among TB patients and for connecting any coinfected patients with HIV services. Although, on the surface, this description implies a clean division of labor, in practice there is substantial overlap and cooperation required, and implementation approaches to achieve this have varied by country [8].

Under the revised Global Plan to Stop TB [9], the key TB/HIV targets (the percent of PLHIV screened for TB, of TB patients tested for HIV, and of eligible people on IPT, cotrimoxazole preventive therapy (CPT), and antiretroviral therapy (ART)) are all 100%. Still, however, global achievements fall far short. In 2010, only 7% of PLHIV were screened for TB, 34% of TB patients knew their HIV status, 0.5% of PLHIV got IPT, and 77% and 46% of identified PLHIV with TB were started on CPT and ART, respectively [3].

Based on an analysis of global and Cambodia-specific literature, possible reasons for this incomplete implementation include the different cultures and parallel structures of TB and HIV/AIDS national programs, and remaining gaps in the evidence base. Here, we use these two themes as a conceptual framework to review Cambodia’s approach to establishing a TB/HIV collaboration. Although roll-out is ongoing, we seek to document and gain a better understanding of the process by which a collaboration is built. This investigation reveals the central role of research, in which successive waves of health research have influenced health policy, implementation and, ultimately, patient outcomes.

### Recognition of a problem

In Cambodia, TB control activities lapsed during the 1970s and into the 1980s, as conflict all but destroyed the infrastructure and human resources needed for a functioning health system. Although TB prevalence is now declining, it remains high at 660 cases/100,000 population/year for 2010 [3]. HIV prevalence among these TB patients is estimated at 6.3% compared to 0.8% amongst the general adult population [10]. A concerted effort to build a TB control system began only in 1993 [11]. Ten years later, Cambodia’s national HIV/AIDS program began providing ART, which is now supplied to 42,034 adults and 4,439 children (~78% of those in need, with “need” defined as all those with CD4 less than 350) [12].

Early HIV/AIDS teams in Cambodia documented and acknowledged TB as the number one opportunistic infection (OI) among PLHIV. Indeed, a coordinating body for TB/HIV has existed for over a decade. But this body has not always been active, and joint leadership was a challenge. This is perhaps not surprising. The National Center for HIV/AIDS, Dermatology and STDs (NCHADS) and the National Centre for Tuberculosis and Leprosy Control (CENAT) operate under different models that are not a natural match: NCHADS relies on fewer, specialized centers (currently 56 OI/ART centers) and a mobile system of home based care, whereas CENAT is more decentralized in delivering services (via 1071 health facilities (TB services), and a set of community DOTS workers distinct from the home based care teams). Bridging this organizational divide was never going to be easy. In addition, relative to NCHADS, CENAT has fewer resources [13], and both programs reflect the verticality present in the international partner landscape.

This challenging scenario makes the recent progress all the more interesting. There has not been a single, all-encompassing policy change; rather, progress has been made via serial, smaller advancements from the TB and HIV sides (Table 1), with each advance encouraging the other program to act next.

**Table 1:** Timeline of TB/HIV activities in Cambodia*

**Year**	**Type**^**^**^	**Initiating agency**	**Intervention**	**Outcome**	**Challenges**
1993	I	CENAT	DOTS expansion initiated	Availability of TB treatment increased	Post-conflict environment
1999	P	MoH	TB/HIV subcommittee formed	Dialogue initiated	Irregular meetings; limited action
2002	P	CENAT and NCHADS	Framework for TB/HIV	First formal agreement	Agreement remained general; technical direction still unclear
2003	I	NCHADS	ART initiated under Continuum of Care; OI/ART team included TB physician	Availability of ART increased	Initially low capacity of healthcare system
2003	R	NCHADS, CENAT, WHO, FHI, US CDC, JICA	TB/HIV pilot programs	Pilots initiated and results published; operational challenges highlighted	Commitment to national roll-out not present prior to pilots, and relative roles of CENAT and NCHADS were not yet defined
2006	P	CENAT and NCHADS	SoP on HIV testing of TB patients and TB screening of PLHIV	Relative roles of CENAT and NCHADS defined; OI/ART teams screened PLHIV for TB and CENAT used 3 options to increase screening of TB patients for HIV	Technical questions remained, i.e., research was needed to define best practices for ART initiation and TB symptom screening prior to IPT
2009	R	CENAT, NCHADS, US CDC, Cambodian Health Committee and research partners	CAMELIA and ID-TB/HIV studies completed and results disseminated in Cambodia	Results define when ART should be started in TB patients and what symptom screen to use in PLHIV prior to IPT	Very few challenges; rapid adoption of findings into field practice
2010	P	TWG for TB/HIV, NCHADS with CENAT	3Is SOP completed	Roll-out of 3Is, based on detailed roles and responsibilities	TB screening of existing PLHIV may put burden on TB diagnostic services
2010	P	CENAT, NCHADS	Revised TB/HIV framework	All TB/HIV policy captured in a single document	

*Abbreviations not defined in main text: MoH (Ministry of Health); FHI (Family Health International); US CDC (US Centers for Disease Control); JICA (Japan International Cooperation Agency); TWG (Technical Working Group).

^Types of activities are research (R), policy (P), and implementation (I).

### Pilots, SOPs and evidence

Early on, the higher levels of the Ministry of Health and global and external organizations drew attention to TB/HIV, and there was critical support for action from the leadership of both CENAT and NCHADS. Four TB/HIV pilots established in Cambodia in 2003 [14] did not transition into joint national scale-up, but they did provide experience and evidence for two advancements. First, in 2006 CENAT and NCHADS signed a standard operating procedure (SOP) for HIV testing of TB patients and TB screening of PLHIV [15]. Though brief, this SOP made the valuable contribution of defining the core competencies and commitments of each program; this was a roadmap for both the programs and for donors and partners. It was clear that neither program could tackle the problem alone. Second, the pilots provided evidence on strategies that worked (such as scripts and data collection forms to encourage TB screening and HIV testing [16] and cost effective approaches to ICF and IPT [17]) and highlighted certain operational questions that needed answering.

Some of these questions were tackled in trials conducted in Cambodia. The CAMELIA (Cambodian Early versus Late Introduction of Antiretroviral Drugs) trial [18], building on the related South African trial [19], established the need to start ART early in coinfected patients. This emphasized the importance of a unified, coincident approach to treatment, rather than the sequential treatment that had been common. In addition, the Improving Diagnosis of TB in HIV-Infected Persons (ID-TB/HIV) study provided a simple protocol for screening PLHIV in order to exclude TB [20], thus paving the way for provision of IPT. Half of the patients for this study came from Cambodia, where the findings were disseminated in 2009. Awareness of these two trials and their results has driven incorporation of the latest international evidence into national policies.

### Options, incentives and experience

TB/HIV visibility and guidance was maintained via regional conferences, frameworks [21] and linkages to meetings that emphasized global normative standards. Meanwhile, the conduct of individual TB/HIV activities was providing experience and influencing policy development. Screening of PLHIV for TB was ongoing, facilitated by the inclusion of a TB physician on most OI/ART teams. For HIV testing of TB patients, CENAT found that referral of blood was a far more efficient option than referral of patients or the use of mobile testing units. Financial enablers to support this blood transport boosted testing rates even further, from ~50% to ~90%, depending on the province, and TB services used their repeat encounters with patients to talk about the importance of testing. These and related CENAT activities were acknowledged by NCHADS as bringing HIV services to health centers, and thus closer to clients. Furthermore, these experiences made development of the more comprehensive SOPs and frameworks both less threatening – because experience had already clarified which activity was being undertaken by which program – and more practical.

Formulation of the SOP for the 3Is was coordinated by the Technical Working Group for TB/HIV, which brought together opinions from NCHADS, CENAT and partners, monitored progress, and maintained pressure for action. This completed SOP [22] includes precise and practical details on relative responsibilities (e.g., CENAT procures isoniazid for NCHADS to carry out IPT), although challenges remain for areas with less clear guidance (e.g., certain recording and reporting activities, and the diagnostic workup of PLHIV who are positive for the TB suspect symptom screen). The revision of the overall TB/HIV framework [23] reflects the new 3Is SOP, which by 2013 will be implemented nationwide.

### Outcomes

In terms of policy, evidence, and experience, the critical building blocks for TB/HIV collaboration in Cambodia are now in place. Financing for TB/HIV activities is also available and allowing for rapid rollout; of note, the Global Fund grant process has, in effect, provided a forum for joint planning.

The timing of roll-out, and thus the outcomes of implementation thus far, varies between the different activities. TB screening of existing OI/ART clients only became a policy in 2010, and is being introduced along with the new symptom-based screening, but already 87% of newly registered OI/ART clients were screened for TB in early 2011. At TB services, HIV testing of TB patients has been in place for several years, so in 2011 82% of notified TB patients had a known HIV test result [24]. With roll-out of the 3Is SOP, 85.5% of coinfected individuals were confirmed as started on CPT and 78% on ART [24]; these numbers built upon a doubling and tripling from 2009 to 2010 due largely to improvements in reporting. Finally, from 2009 to 2011 the provision of IPT has expanded significantly (from a coverage of 66 PLHIV without TB in 2009 to 645 in 2011), and should continue to rise with the 3Is roll-out and new screening algorithm.

## Conclusions

Cambodia’s path to TB/HIV implementation has not been easy. Consistent with the framework for this article, there have been challenges in overcoming the separation between the TB and HIV programs to address the first step – the establishment of a politically powerful and active mechanism for coordination, which requires the cooperation of both programs. A major cause of these challenges appears to have been the early gaps in the evidence base and thus incomplete technical guidance. But the country’s progress illustrates that these challenges need not prevent progress. Instead, the conduct of critical research and the gradual definition of each program’s activities can lead, eventually, to greater trust, a better understanding of how the different implementation approaches driving the TB and HIV/AIDS national programs can complement each other, and a more comprehensive response to the TB/HIV challenge. Two programs, strong individually, have built upon each other’s advances, and the processes of policy, practice and research have reinforced each other to keep the TB/HIV agenda moving. In providing this example, Cambodia’s TB/HIV activities will, if done well, have an impact well beyond the country’s borders.

## Competing interests

The authors declare that they have no competing interests. The views expressed in this paper are those of the authors and do not necessarily represent the positions of their respective organizations.

## Authors’ contributions

MTE and MCV guided the development of TB/HIV collaborative activities in Cambodia and oversaw the current review process; RY, MF and BT formulated the review process; KKE, RY, MF and WW collected field information as background for the review; WW analyzed the field information and drafted the text; and all authors critically revised the text and approved the final manuscript.

## Authors’ information

MTE and MCV are national program managers of the National Centre for Tuberculosis and Leprosy Control (CENAT) and National Center for HIV/AIDS, Dermatology and STDs (NCHADS), respectively. KKE is Vice Chief of Technical Bureau and Focal Point for National TB/HIV Collaboration at CENAT; SS, SS, and NB are Chief of AIDS Care Unit, Deputy Chief of Technical Bureau, and Deputy Chief of AIDS Care Unit at NCHADS. RY and MF are Medical Officers for TB and HIV, respectively, at WHO Cambodia. BT is Technical Officer, Global Health Partnerships, at WHO Western Pacific Regional Office (WPRO); MG and PvM were formerly HIV and TB regional advisers, respectively, for WHO WPRO; MG is now HIV regional adviser for PAHO, and PvM is the WHO representative for WHO Cambodia. WW is an independent consultant.
